# Supplementary figures and images for: Prioritized candidate causal haplotype blocks in plant genome-wide association studies
Source: PLoS Genet. 2022 Oct 17;18(10):e1010437. doi: 10.1371/journal.pgen.1010437 (PMC9612827; doi:10.1371/journal.pgen.1010437)

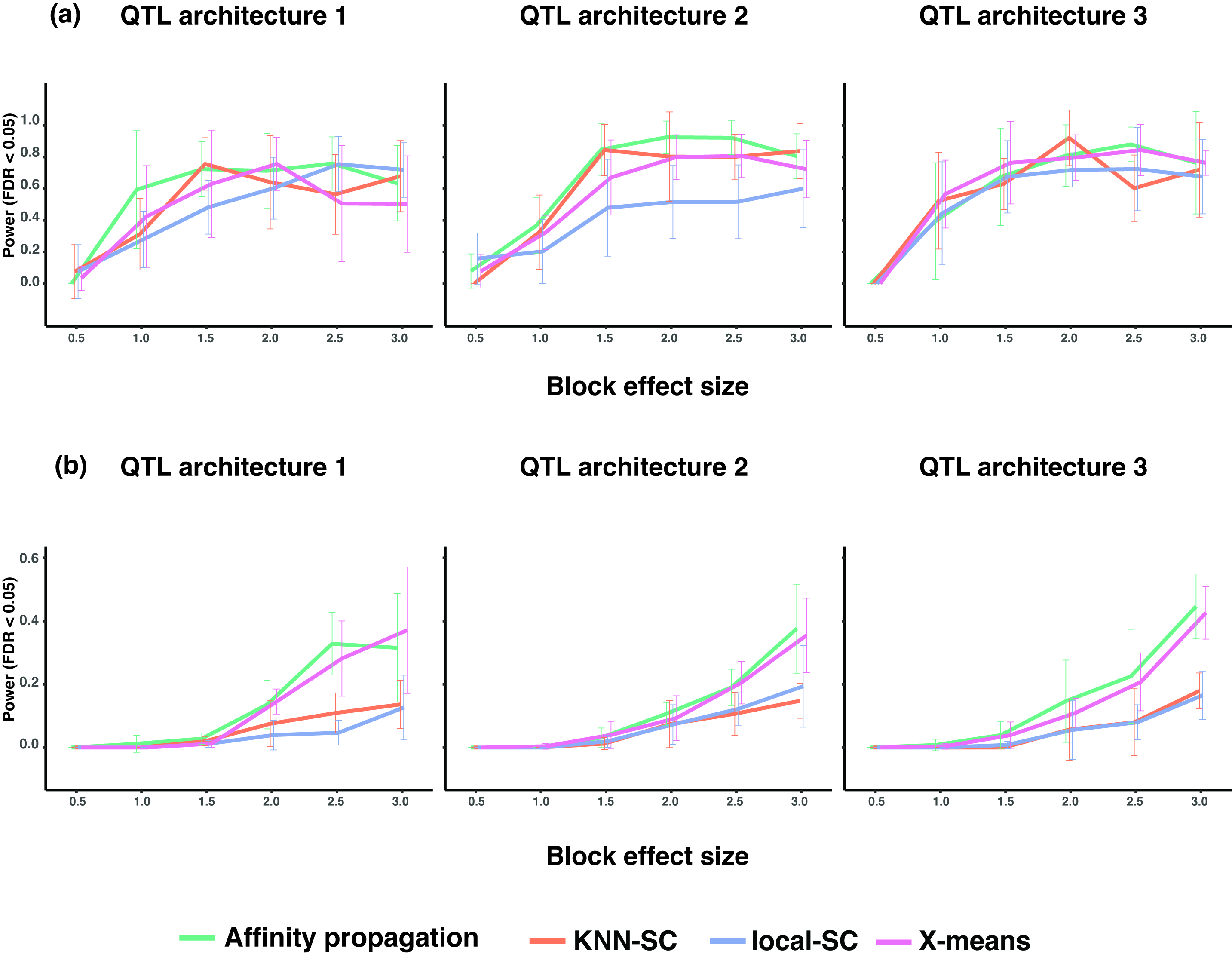

Supplement: S1 Fig — (a) Mapping power (FDR < 0.05) of affinity propagation, KNN-spectral clustering, local-spectral clustering and X-means in the low polygenicity simulation. (b) Mapping power (FDR < 0.05) of affinity propagation, KNN-spectral clustering, local-spectral clustering and X-means in the high polygenicity simulation. (TIF) [file pgen.1010437.s001.tif]

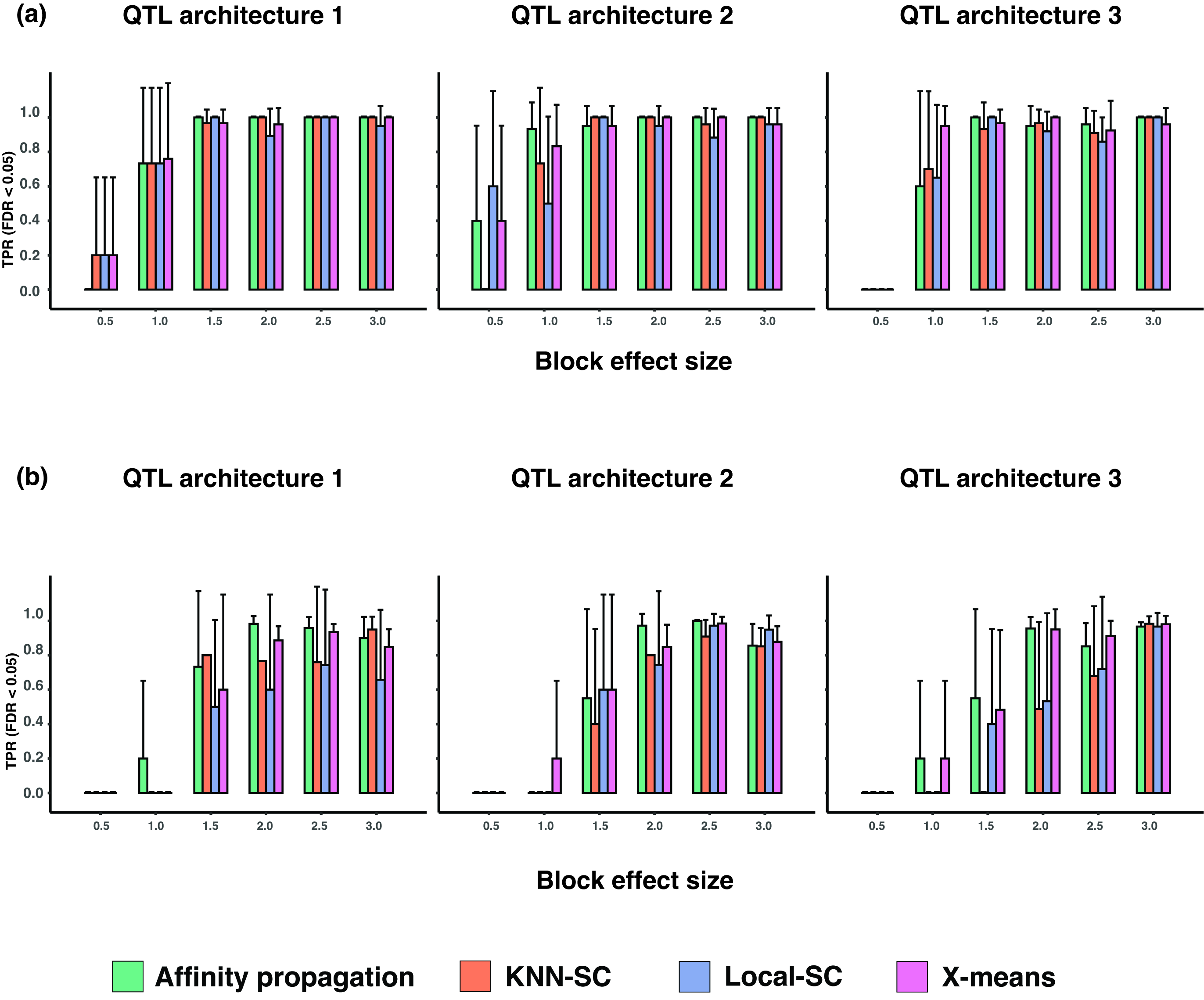

Supplement: S2 Fig — (a) TPR (FDR < 0.05) of affinity propagation, KNN-spectral clustering, local-spectral clustering and X-means in the low polygenicity simulation. (b) TPR (FDR < 0.05) of affinity propagation, KNN-spectral clustering, local-spectral clustering and X-means in the high polygenicity simulation. (TIF) [file pgen.1010437.s002.tif]

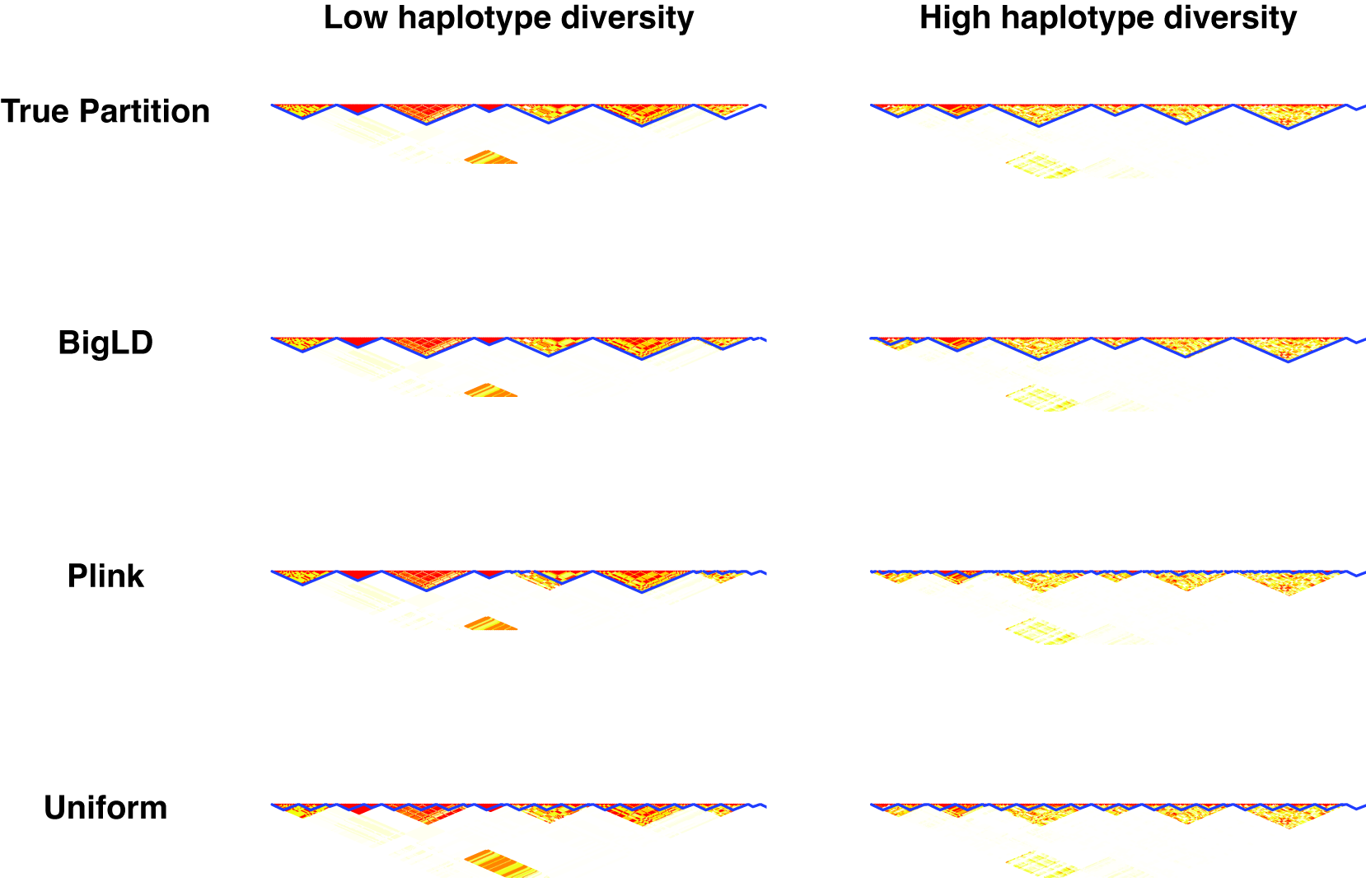

Supplement: S3 Fig — Each block partition algorithm was tested on low haplotype diversity and high haplotype diversity simulations. The redness indicates the strength of LD between SNP pairs, and the blue line indicates the block partition generated by the method. (TIF) [file pgen.1010437.s003.tif]

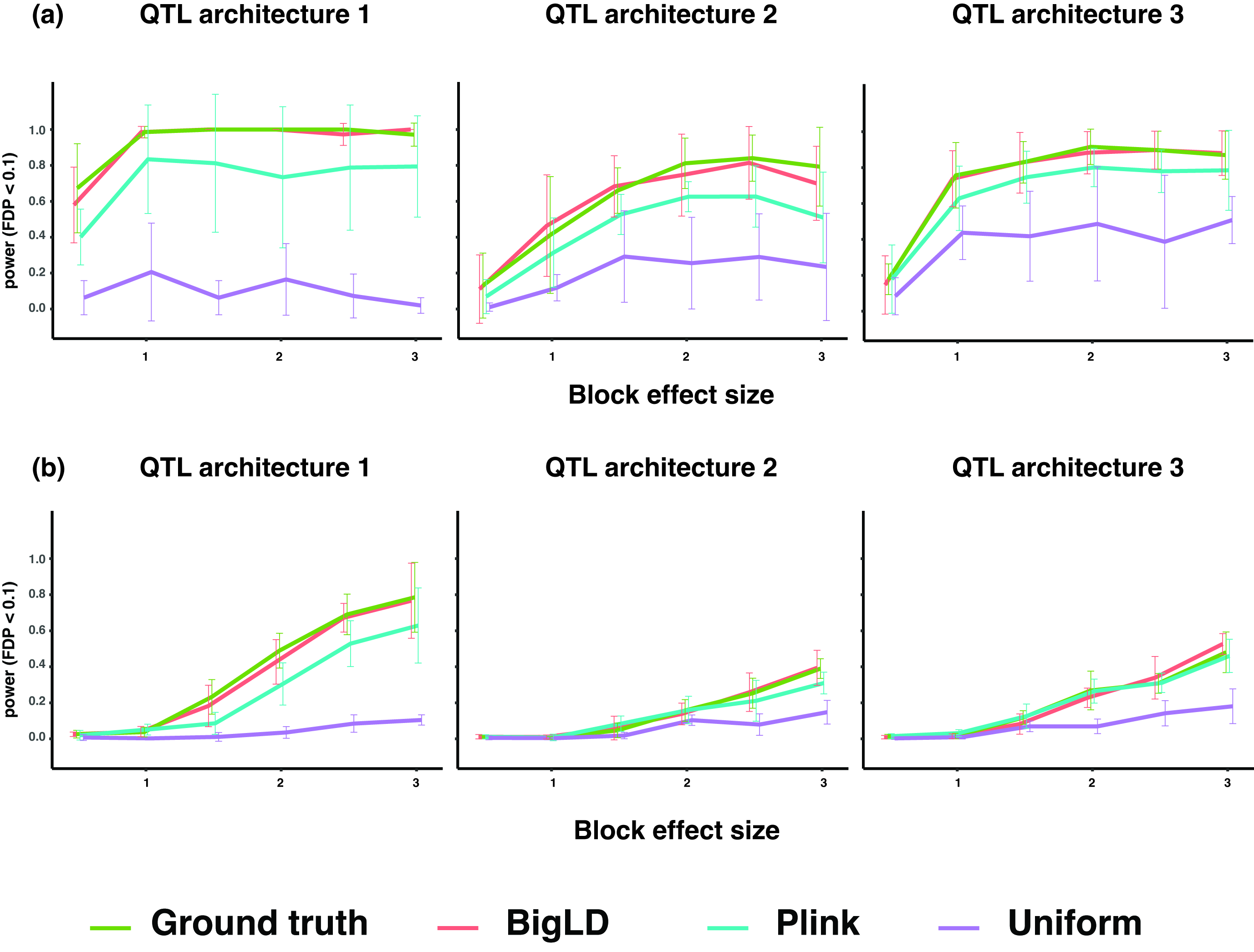

Supplement: S4 Fig — The x-axis indicates the per-locus heritability. (b). Mapping power comparison (FDR < 0.05) of block partition algorithms in the low haplotype diversity and low polygenicity simulations. (c). Mapping power comparison (FDR < 0.05) of block partition algorithms in the high haplotype diversity and low polygenicity simulations. (TIF) [file pgen.1010437.s004.tif]

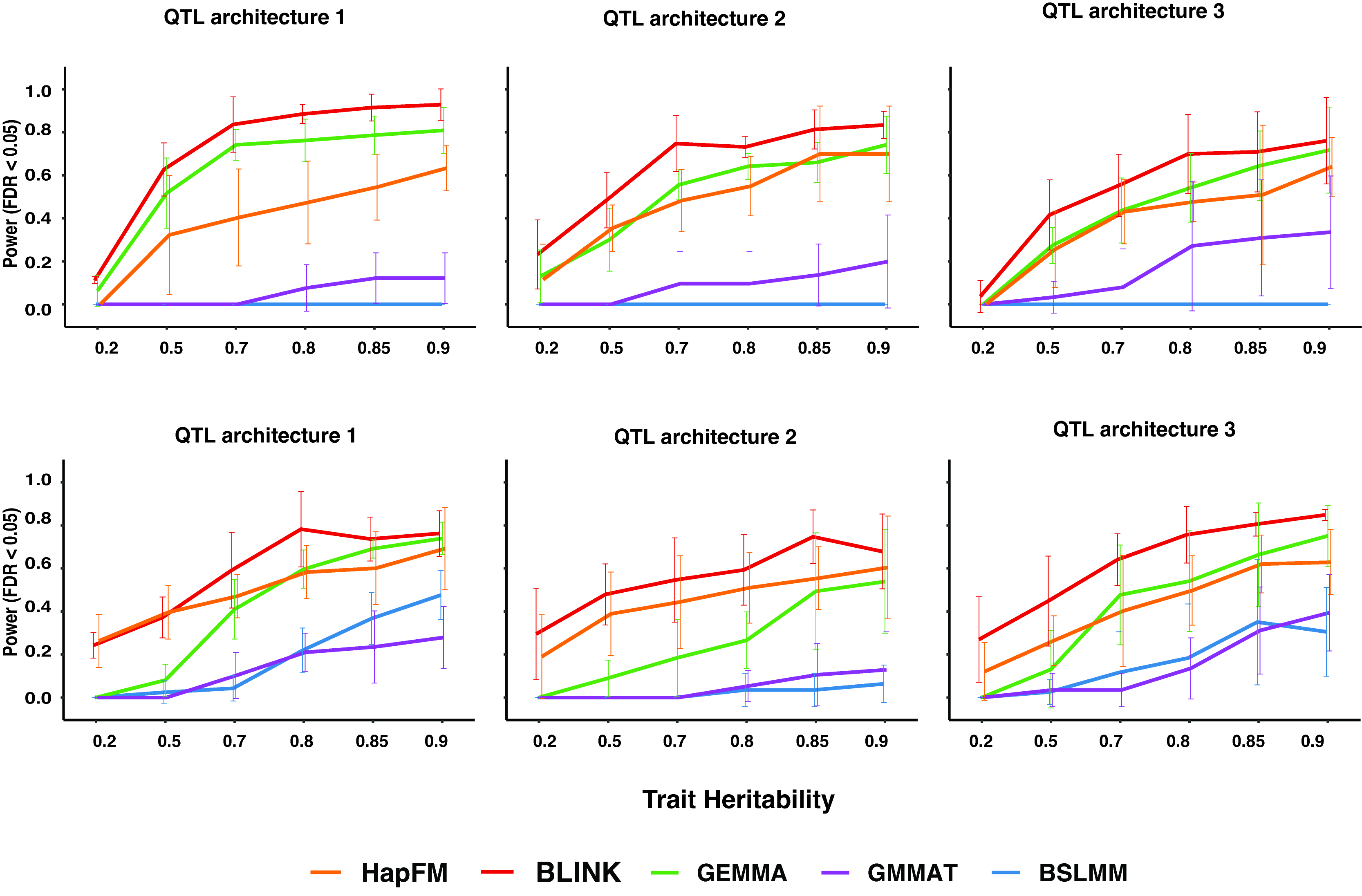

Supplement: S5 Fig — The x-axis indicates the per-locus heritability. (a). Mapping power comparison (FDR < 0.05) of different GWAS methods from the Arabidopsis dataset containing 1135 individuals. (b). Mapping power comparison (FDR < 0.05) of different GWAS methods from the soybean dataset containing 2898 individuals. (TIF) [file pgen.1010437.s005.tif]

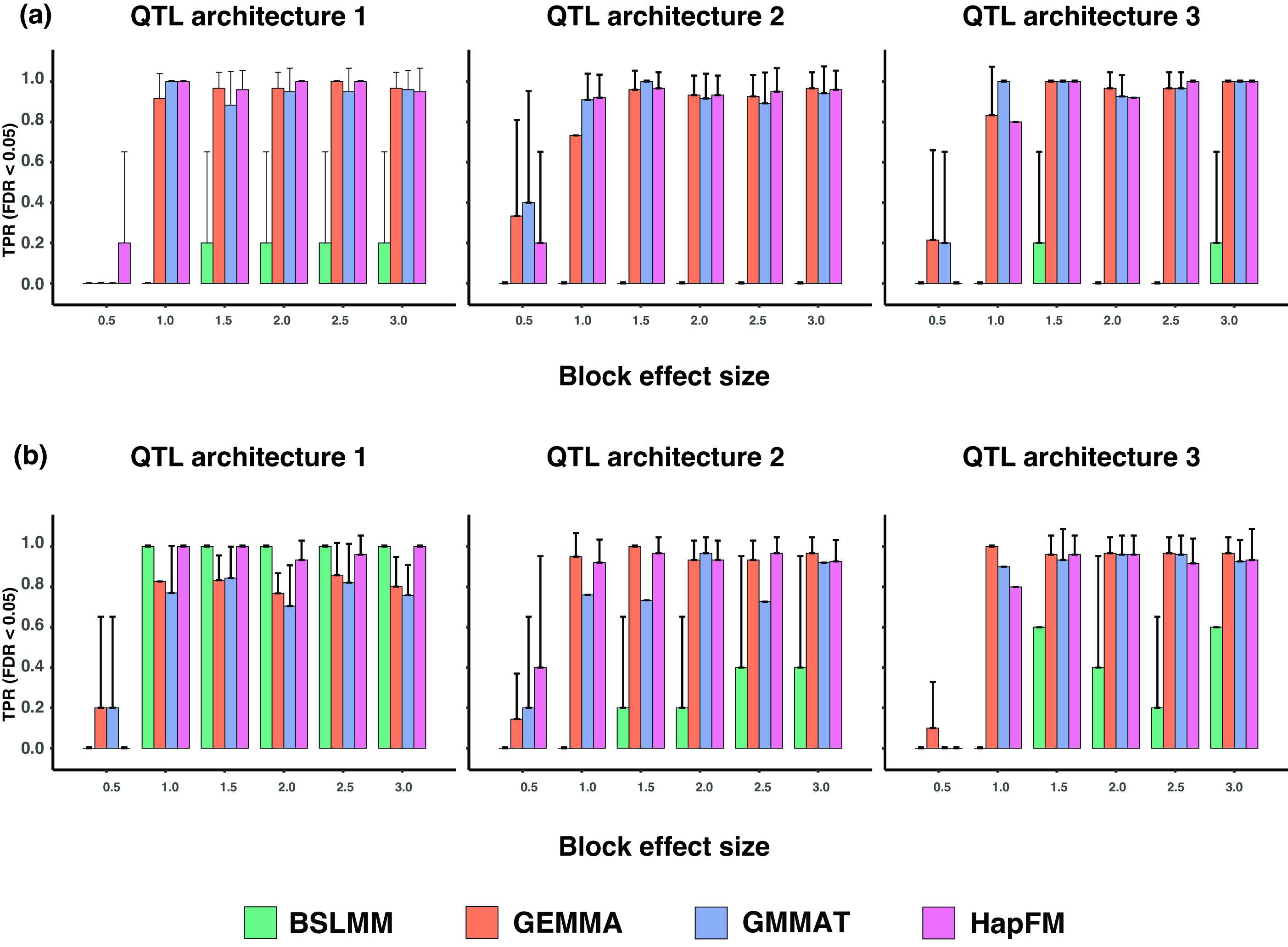

Supplement: S6 Fig — (a). True positive rate (FDR < 0.05) of different GWAS algorithms from the Arabidopsis simulated dataset. (b). True positive rate (FDR < 0.05) of different GWAS algorithms in the soybean simulated dataset. (TIF) [file pgen.1010437.s006.tif]

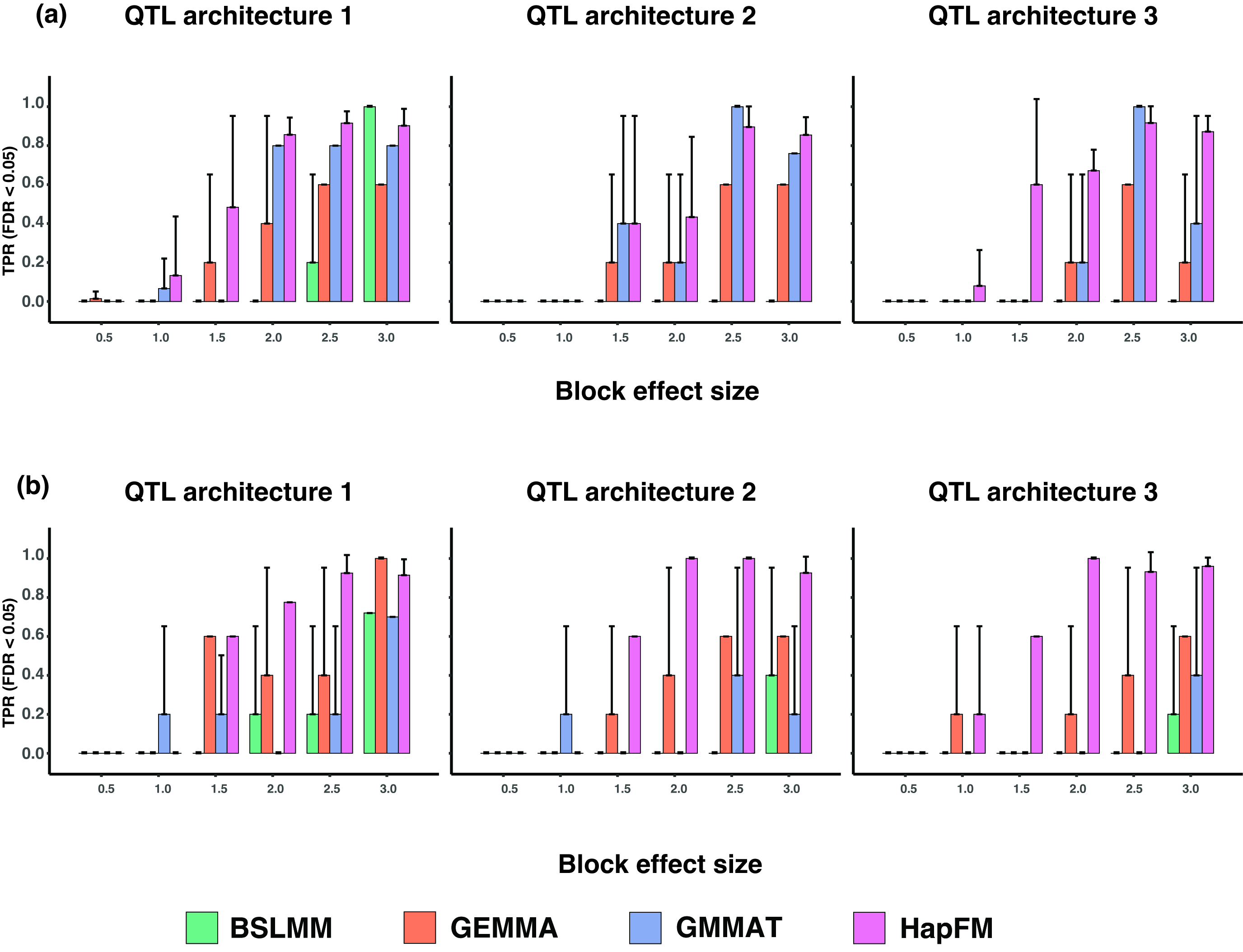

Supplement: S7 Fig — (a). True positive rate (FDR < 0.05) of different GWAS algorithms from the Arabidopsis dataset. (b). True positive rate (FDR < 0.05) of different GWAS algorithms from the soybean dataset. (TIF) [file pgen.1010437.s007.tif]

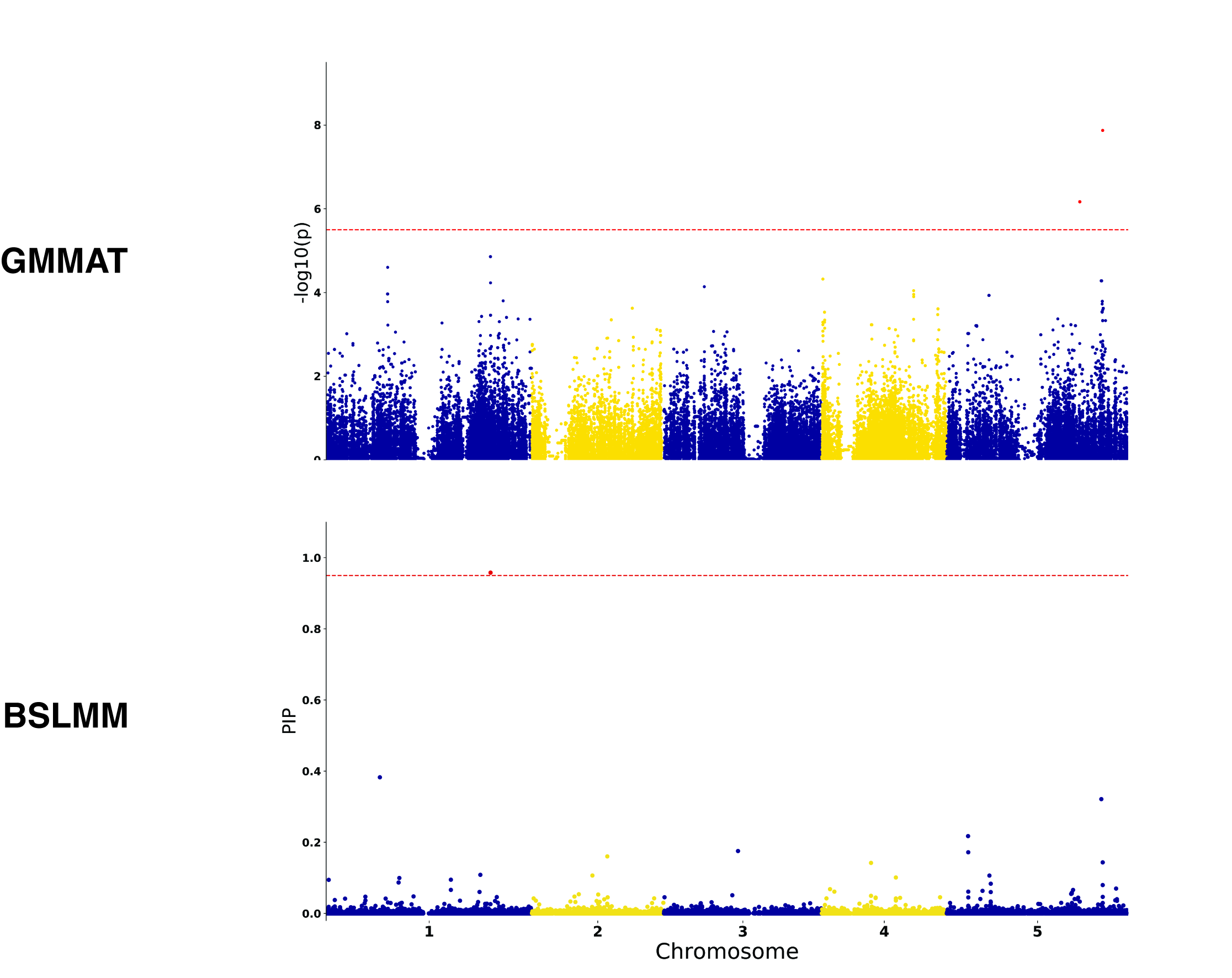

Supplement: S8 Fig — The red dash line indicates the FDR 0.05 threshold. (TIF) [file pgen.1010437.s008.tif]
