# Supplementary material for: Prioritized candidate causal haplotype blocks in plant genome-wide association studies
Source: PLoS Genet. 2022 Oct 17;18(10):e1010437. doi: 10.1371/journal.pgen.1010437 (PMC9612827; doi:10.1371/journal.pgen.1010437)
Supplement: Attachment — Submitted filename: editor_comments.docx [file pgen.1010437.s011.docx]

**Comments to the Authors:**

Reviewer #1: 1. L62-63: The study mentioned the variation of marker-based association methods, including MLMM and FarmCPU. These two methods are in a different category from the other conventional maker-based association methods. They use a multiple loci model where associated markers are included as covariates to improve statistical power. There are even more development multilocus models (e.g. BLINK). Although the study benchmarked several GWAS methods, including GEMMA, BSLMM, and SMMAT, the multilocus models are completely missing.

BLINK is now included in the revised manuscript. It was benchmarked against other GWAS methods in both simulated and real datasets.

2. L471: Add details of genotype simulation for the 500 individuals, such as initial individuals, number of generations, population size, and mating. Also discuss why not using real genotypes.

There are two simulation analyses now in the revised manuscript. The first simulation was done using the synthetic diverse population which contained 500 individuals. The purpose of using this synthetic population was to benchmark the performance of different block partition and haplotype clustering algorithms. The second simulation was based on real Arabidopsis and soybean genotypes to benchmark the performance of different GWAS algorithms.

3. L488-491: In addition to causal SNPs in blocks, blocks were also assigned with effects. What are their genetic bases if they are not SNPs, structure variation?

The genetic basis of effect of a causal block can be the combined additive and epistatic effects of causal variants in the block. The effects were assigned in a hierarchical manner in the simulation studies. The block effect was equal to the sum of the effects of the causal SNPs in the block because we only considered additive effects in the simulation. There could be more than one causal SNPs in the causal block, therefore, we fixed the effect size of each causal block and then assign effects to the causal SNPs in an additive manner based on how many causal SNPs were simulated inside the block.

4. L491-497: The total number of causal SNPs is less than ten, which is far away from reality containing hundreds or thousands of genes.

In every causal block, we simulated different number of causal SNPs, from one to six in most cases. However, there are hundreds of causal blocks genome wide. Therefore, there are hundreds or thousands of causal SNPs in total.

5. Is HapFM only a pipeline concept described by Figure 1 for users to code by themselves, or a software users can analyze data directly, or both?

HapFM is now publicly available on github. Please see line 40 in the revised manuscript.

Reviewer #2: The authors developed a haplotype-based GWAS approach called HapFM. They used simulated and real data in Arabidopsis, rice, cassava, and maize to test this approach, and to compare its performance with commonly used SNP-based GWAS approaches. They found that for traits that are polygenic, HapFM outperformed other tested SNP-based GWAS approaches. Importantly, they also found that HapFM had better resolution in high-LD regions

I think that the analysis conducted in this paper is solid. However, I think that some additional refinement to the study is needed, the manuscript itself needs some polishing, and some careful editing to how this work is presented and introduced to the plant community is needed. If these suggestions below are considered when updating and revising this paper, I think that HapFM has potential to be widely used in the plant/crop GWAS community.

Major Points
1.) The word “casual” implies that there is a definitive, proven, biological association between a genomic locus and a trait. No matter how sophisticated a GWAS approach is (i.e., even if the degree putative causality is included in the prior), a GWAS is a series of statistical analyses, and will never be able to definitively prove the existence of a causal mutation. I suggest toning down the claim that what is being identified with HapFM is something casual, unless a bona fide biological analysis (e.g., molecular or functional biology) can confirm causality.

The causal word was taken from the field of statistical inference. We agree with the reviewer that the word “causal” may confuse the plant community with the functional causality of the variants. We have changed the wording to prioritize candidate causal regions. The title of the manuscript has changed to “prioritize candidate causal haplotype blocks in plant GWAS studies”.

2.) Please put the Materials and Methods before the Results section. Doing so will make the implications of the work presented in the Results section more meaningful.

The Material & Methods section is now before the Results section in the revised manuscript.

3.) Was the false positive detection rate of HapFM explored? If not, I suggest simulating a setting where the heritability of the trait is 0, and seeing how often HapFM detects false positives.

The false positive detection rate was evaluated in the supplemental figure 6-7 in the revised manuscript.

4.) Please clarify what is meant by “mapping interval length”. Does this refer to LD decay surrounding an identified SNP of interest from GEMMA? Although I completely agree that interval length is important, I will say that the term “interval length” is not commonly used in plant/crop GWAS. Thus, putting in an extra effort to precisely define this term as early as possible in the manuscript will help the plant/crop GWAS community understand this important concept more clearly.

Suggestion taken. The definition of mapping interval length is included in the introduction section of the revised manuscript. Please see line 75 in the revised manuscript.

5.) I suggest carefully revising the first paragraph of the Discussion. The plant and crop community have been conducting GWAS for over two decades, and researchers have come up with a lot of innovative approaches to overcome the unique challenges posed by crop data sets. I can see, for example, plant and crop genetics researchers taking exception to comments like “…a dearth of suitable genomic datasets” (Line 279-280). While it is true that realities of collecting diversity panels for plant/crop GWAS results in substantially smaller sample sizes than in human data sets, association studies have been able to identify important associations that have advanced both basic biology and crop breeding research. I agree that HapFM is an excellent approach, and I can’t wait to try it out for GWAS in crops and plants, but I think that the tone taken in the first paragraph of the Discussion could dissuade the plant and crop GWAS community from trying this approach.

Suggestion taken. The first paragraph of the discussion was rewritten in the revised manuscript. Please see line 464-476.

Minor Points

I am noticing minor little typos throughout the manuscript, but nothing that another round of careful editing cannot fix.

Lines 63-65: I agree with this sentence, but suggest providing a reference to back up this statement. Also, it is also worth noting that the marker sets typically used in plant GWAS are highly unlikely to include the causal mutations underlying traits. I suggest adding this somewhere to the Introduction.

Suggestion taken. Very good advice. Please see line 64 in the revised manuscript.

Line 65: I suggest replacing “variants” with “markers” to make it clear that GWAS is conduct on marker sets, and it is highly likely that causal variants are not included in the marker sets.

Suggestions taken.

Line 67: please introduce the words to of the acronym SMMAT. This same comment applies to RAINBOW (Line 69) and FH-GWAS (Line 70)
Suggestion taken. Please see line 68 in the revised manuscript.

Lines 131-133. I have two comments: i.) please provide more details about the LMMs, and please be sure to cite previous work that has used LMMs (e.g., the unified mixed linear model from Yu et al. 2006, <https://doi.org/10.1038/ng1702>). ii.) Please describe what fixed and random effects are being included in the model to account for spurious associations.

Suggestion taken. The details about the statistical model was stated in the material and method part. Please see line 145 in the revised manuscript. The references of LMM were included in the line 63.

Lines 133-135: How does knowing LD between haplotype blocks alone provide definitive proof of a causal association? I suggest editing this sentence so that the word “causal” is not included (please see my first major point about causality).
Suggestion taken. The wording has changed to prioritize candidate causal blocks. Considering LD in the mapping model allows user to prioritize blocks.

Lines 164-166: please provide a definition of power within the context of these simulations.

Suggestion taken. Please see line 279.

Lines 162-163: Please define exactly what is meant by “major” and “minor” effects? Also, were these effect additive, dominant, or epistatic (and if the latter, which type of epistasis was simulated)?

Suggestion taken. The words major and minor were changed to large and small. All the simulated the effects were additive. Please see line 345 in the revised manuscript.

Line 175: Somewhere in this paragraph, I suggest describing how many SNPs were considered in these studies. With respect to multiple testing correction, I would imagine that SNP-based GWAS approaches will have many more hypothesis tests to correct for than HapFM.

Suggestion taken. Please see line 361-385 in the revised manuscript. Yes, SNP-based GWAS methods have more hypothesis tests than HapFM.

Line 179: Instead of calling it “QTL architecture 2”, describe the genetic architecture here. This will prevent the reader to having to go back to Figure 2 to remember what “QTL architecture 2” refers to. This same comment applies to similar subsequent instances (e.g., Lines 183-185).

Suggestion taken. Detailed description about different QTL architectures were included in the revised manuscript in the material and methods sections. Please see line 206 in the revised manuscript.

Line 282:- Please elaborate on what is meant by “underpowered”.

Suggestion taken. Please see line 476-477

Line 314: Technically speaking, GEMMA, GAPIT, and FarmCPU are software, as well as GWAS approaches (and GAPIT is strictly an R package, which implements multiple GWAS approaches). I suggest refining the terminology in this sentence.

Suggestion taken. Please see line 509 in the revised manuscript.

Line 321-322: The multiple testing problem will still exist for HapFM, right? HapFM still involves multiple hypothesis tests, and there will still be a need to adjust for multiple testing.

Yes, multiple testing problem still exists technically. However, HapFM employs the full hierarchical Bayesian model and uses MCMC for parameter inference. The prior probability models null effect and true effect, and in the end posterior inclusion probability (PIP) of each haplotype will be reported, which have already accounted for multiple testing. HapFM will automatically convert PIP to false discovery rate (FDR) for easier interpretation.

I the figure captions provided the ideal amount of detail of describing the figures. Thank you for putting in the effort to write excellent captions!

Reviewer #3: The submitted manuscript present an algorithm to improve the power of identifying candidates genes in plant ppopulations. The motivation is outlined by comparison of GWAS succcess in human genetics with larger sample sizes, to that of plant genetics, with smaller smaple sizes and strong population structure. The manuscript outlines the idea of using LD-informed haplotype blocks instead of SNPS to both remove reliance on tag-SNPs and reduce the multiple testing burden. Finally, the algorthim is incorporated with fine-mapping to reduce QTL intervals. This agorithm, HapFM, is compared to other GWAS algorthims across both simulated and empirical datasets.

The manscript is well structured, and provides a thorough discussion.

General Comments:

1. I disagree with the language of causality used throughout the manuscript. Upon reading, all methods compared results in statistical inferences of effect sizes on traits and mapping intervals. The only link to causality is possibly through candidates identified in previous studies. Not sure if these were ever confirmed for causality. I think the title and manuscript should be reframed or reworded to reduce the stress on causality. For example L134-135: "The fine-mapping model accounts for the LD between haplotype blocks, and therefore the result suggests the causal instead of association relationship with the phenotype." - How? All of this analysis is associative? (Not a problem in itself)

Suggestion taken. We agreed that the wording is confusing, we have changed that to prioritize candidate causal blocks in the revision manuscript.

2. The datasets used in this study:
The manuscript articulates that new GWAS methods are required to account for small population sizes and high LD ofd plant population that have been under selection during modern breeding. However I question the relevance of the datasets employed for the comparison of the methods.

We simulated small diverse GWAS populations because many plant GWAS populations were small due to many reasons. We tried to simulate more realistic datasets in plant GWAS research. In order to mimic a realistic plant GWAS dataset, we included the real plant-genotype-based simulation in the revised manuscript.

Empirical Datasets: At least some of the datasets, e.g. Arabidopsis 1001, resemble something closer to a diversity panel. Rather than populations tht have undergone modern breeding.

We wanted to select diverse populations rather than mapping population that have undergone modern breeding.

Simulated Datasets: From the Methods section it is hard to understand how the genomes simulated relate to readily developed plant populations. There no mention of pedigree structure, whether this population has undergone selection or not and reached Bulmer equilibrium. These points are critical in the development of realistic LD patters across the genome and its influence on the genetic variance of the simulated traits.

There are now two simulations in the revised manuscript. The first simulation was pure statistical simulation of genotypes to evaluate different block partition and haplotype clustering algorithms. The second simulation was based on real plant genotype data from1135 Arabidopsis individuals and 2898 soybean individuals. This simulation was used to benchmark different GWAS methods on their mapping power, mapping interval length and true positive rate.

The manuscript would benefit from clearer articulation of the LD patterns in these datasets and the traits studies to strengthen the link to application in crop improvement. Without this, its hard to understand if this algorthim will provide improved GWAS results and candidate gene identification in relevant mapping and breeding populations.

Specific Comments:

L31-32: "infer the causal haplotype blocks of trait." - I don’t think this statement can be supported with the detail currently provided in the manuscript.

Suggestion taken. Please see line 31 in the revised manuscript.
L121: Reference or name the available block partition algorithms

Reference is now included in the revised manuscript. Please see line 128-129 in the revised manuscript.

L169-170: "Major mapping power differences were found between BigLD and Plink blocks in the high haplotype diversity simulations": It’s important to connect “diversity simulation”; to readily used populations in plant breeding/genetics. Just adding

Suggestion taken. Adding in the discussion section. Please see line 578 in the revised manuscript.

L191-192: "HapFM consistently resulted in the highest mapping power in all four QTL architectures in both low and high haplotype diversity simulations (Figure 3).": Is this due to using haplotypes or incorporation of biological priors, or both? expand in the discussion.

The consistent highest mapping power was due to using haplotypes that aggregates individual variant effects and reduced number of variables. More explanation is now included in the revised manuscript.

Figure4: Interval lengths don’t seem to much reduced apart from GEMMA algorithm.

With the real plant genotype-base simulation, HapFM greatly reduced mapping interval length in all settings. Please see line 387-397 in the revised manuscript.

L211-212: It would be helpful to give brief information on these populations. Are they mapping populations from closely related individuals or diverse accessions. Has intercrossing been performed? I presume not..

The population was simulated as diverse population for GWAS analysis. Clarification is included in the revised manuscript. Please see line 191 in the revised manuscript.

L279-280: "Its utility in plant studies has been limited by a dearth of suitable genomic datasets": I disagree. Yes, sample sizes are smaller and relatedness is generally higher than human genomic datasets. Ne is always going to be small in a lot of crop populations. Therefore, I think there is greater room for better methods (thanks for providing another option), than improving genomic datasets.

Suggestion taken. Very good point. The sentences were changed in the revised manuscript. Please see line 466-479 in the revised manuscript.

L318-319: "This can be problematic since the selection of the representative SNP is arbitrary involving choosing parameters for LD cutoff and physical distance": Haplotype methods don’t overcome this. HapFM just uses different LD cutoffs....

Yes, HapFM relies on high LD cutoffs to break down large independent LD blocks into smaller LD blocks. The small LD blocks are not independent to each other. However, HapFM uses the full model that incorporates the LD information between small LD blocks, which can result in a prioritized candidate causal block for the trait of interest.

Because HapFM doesn’t rely on representative SNPs, it considers all the haplotypes together in the full model, in this sense, it has considered all the SNPs.

L341-342: haplotype fine-mapping and statistical fine-mapping are both statistical.....not causative. Can you provide a better wording/explanation?

Suggestion taken. We agree that the word causal was confusing here. We changed it to prioritizing candidate causal blocks.
