# Supplementary material for: Prioritized candidate causal haplotype blocks in plant genome-wide association studies
Source: PLoS Genet. 2022 Oct 17;18(10):e1010437. doi: 10.1371/journal.pgen.1010437 (PMC9612827; doi:10.1371/journal.pgen.1010437)
Supplement: Attachment — Submitted filename: editor_comments_secondrevision_final.docx [file pgen.1010437.s012.docx]

Dear Editor:

The second reviewer raised a question about whether HapFM has inflated false positive rate when the underlying true effect is 0. This is a very valid concern about the performance of HapFM. We greatly appreciated the critical thinking and addressed this concern by simulating 0-heritability phenotypes using Arabidopsis and soybean genotype datasets. The results indicate HapFM, the same as BSLMM, GEMMA, and GMMAT, does not have an inflated false positive rate at 0-effect loci. The results are shown in the Supplemental Table 1.

The following is reviewer’s comments and our response:

Reviewer #2: I thought that a nice job was done with addressing the reviewers’ comments. I especially appreciate the revision of the first paragraph of the Discussion. My only major comment applies to making sure that the false positive rates of HapFM and the other competing approaches are reported in the manuscript. This point is elaborated on the point below (which I wrote while looking through the revised manuscript):

I went to Supplementary Figures 6-7, and I did not see any reporting of a false positive rate. Presumably, a false positive rate would be the proportion of statistically significant associations at when the block effect size is zero (i.e., X-coordinate of 0.0). Please include the false positive rates in these figures, and please report the false positive rates in the manuscript. If HapFM is yielding equal or greater true positive rates than other approaches, yet is also yielding a higher false positive rate than theoretically expected, then the reader needs to know about this.

Response: We had inadvertently omitted this data. False positive rate for each GWAS algorithm was tested by simulating 0-heritability phenotypes using Arabidopsis and soybean genotype datasets. The results are now shown in the Supplemental Table 1. In the manuscript, these results are described from Line 398 to 402. In conclusion, HapFM does not inflate false positive rate when the underlying true effect is 0.

Sincerely yours,

Stephen L. Dellaporta

Professor of MCD Biology
